# Testing the implementation and sustainment facilitation (ISF) strategy as an effective adjunct to the Addiction Technology Transfer Center (ATTC) strategy: study protocol for a cluster randomized trial

**DOI:** 10.1186/s13722-017-0096-7

**Published:** 2017-11-17

**Authors:** Bryan R. Garner, Mark Zehner, Mathew R. Roosa, Steve Martino, Heather J. Gotham, Elizabeth L. Ball, Patricia Stilen, Kathryn Speck, Denna Vandersloot, Traci R. Rieckmann, Michael Chaple, Erika G. Martin, David Kaiser, James H. Ford

**Affiliations:** 1 RTI International, 3040 E. Cornwallis Rd., P.O. Box 12194, Research Triangle Park, NC 27709-2194 USA; 2 School of Medicine and Public Health, University of Wisconsin–Madison, 1930 Monroe St., Madison, WI 53711-2027 USA; 3 Roosa Consulting, Beacon, NY 13224 USA; 4 Department of Psychiatry, VA Connecticut Healthcare System, Yale University, 950 Campbell Avenue (116B), West Haven, CT 06516 USA; 5 School of Nursing and Health Studies, University of Missouri-Kansas City, 2464 Charlotte St., Kansas City, MO 64108 USA; 6 University of Nebraska Public Policy Center, 215 Centennial Mall South, Suite 401, Lincoln, NE 68588 USA; 7 Vandersloot Training & Consulting, 11845 NW Stone Mt. Lane, #108, Portland, OR 97229 USA; 8 School of Medicine Psychiatry, and Greenfield Health Medicine, Oregon Health & Science University, 9450 SW Barnes Road St. 100, Portland, OR 97225 USA; 9 National Development and Research Institutes, Inc, 71 West 23rd Street, New York, NY 10010 USA; 10 Rockefeller Institute of Government, State University of New York, New York, USA; 11 Department of Public Administration and Policy, Rockefeller College of Public Affairs and Policy, University at Albany, 1400 Washington Avenue, Milne 300E, Albany, NY 12222 USA

**Keywords:** Implementation strategies, External facilitation, Type 2 hybrid trial

## Abstract

**Background:**

Improving the extent to which evidence-based practices (EBPs)—treatments that have been empirically shown to be efficacious or effective—are integrated within routine practice is a well-documented challenge across numerous areas of health. In 2014, the National Institute on Drug Abuse funded a type 2 effectiveness–implementation hybrid trial titled the substance abuse treatment to HIV Care (SAT2HIV) Project. Aim 1 of the SAT2HIV Project tests the effectiveness of a motivational interviewing-based brief intervention (MIBI) for substance use as an adjunct to usual care within AIDS service organizations (ASOs) as part of its MIBI Experiment. Aim 2 of the SAT2HIV Project tests the effectiveness of implementation and sustainment facilitation (ISF) as an adjunct to the Addiction Technology Transfer Center (ATTC) model for training staff in motivational interviewing as part of its ISF Experiment. The current paper describes the study protocol for the ISF Experiment.

**Methods:**

Using a cluster randomized design, case management and leadership staff from 39 ASOs across the United States were randomized to receive either the ATTC strategy (control condition) or the ATTC + ISF strategy (experimental condition). The ATTC strategy is staff-focused and includes 10 discrete strategies (e.g., provide centralized technical assistance, conduct educational meetings, provide ongoing consultation). The ISF strategy is organization-focused and includes seven discrete strategies (e.g., use an implementation advisor, organize implementation team meetings, conduct cyclical small tests of change). Building upon the exploration–preparation–implementation–sustainment (EPIS) framework, the effectiveness of the ISF strategy is examined via three staff-level measures: (1) time-to-proficiency (i.e., preparation phase outcome), (2) implementation effectiveness (i.e., implementation phase outcome), and (3) level of sustainment (i.e., sustainment phase outcome).

**Discussion:**

Although not without limitations, the ISF experiment has several strengths: a highly rigorous design (randomized, hypothesis-driven), high-need setting (ASOs), large sample size (39 ASOs), large geographic representation (23 states and the District of Columbia), and testing along multiple phases of the EPIS continuum (preparation, implementation, and sustainment). Thus, study findings will significantly improve generalizable knowledge regarding the best preparation, implementation, and sustainment strategies for advancing EBPs along the EPIS continuum. Moreover, increasing ASO’s capacity to address substance use may improve the HIV Care Continuum.

*Trial registration* ClinicalTrials.gov: NCT03120598.

**Electronic supplementary material:**

The online version of this article (10.1186/s13722-017-0096-7) contains supplementary material, which is available to authorized users.

## Background

### Background and rationale for the implementation and sustainment facilitation experiment

Improving the extent to which evidence-based practices (EBPs)—treatments that have been empirically shown to be efficacious or effective—are integrated within routine practice is a well-documented challenge across numerous areas of health [1–5]. A comprehensive systematic review of studies on the costs and efficiency of integrating HIV/AIDS services with other health services noted, “Unfortunately, few of the studies found adequately address the central questions currently concerning many program managers at this moment in time: not whether to integrate, but when to, how to and which model is most efficient in which setting?” [6]. The need to address these central questions about the integration of substance use disorder (SUD) services within HIV care settings is particularly pressing, given the high prevalence of substance use [7–9] and associated problems among individuals living with HIV/AIDS [10–17].

**Fig. 1:**
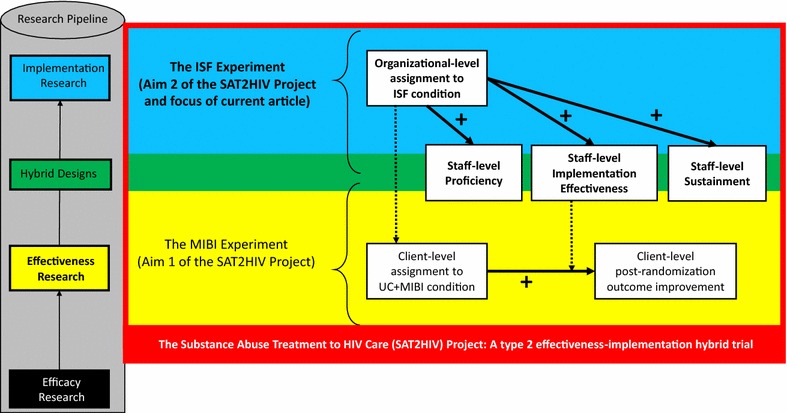
Conceptual overview of the ISF experiment within the context of the parent SAT2HIV Project. *Note*: MIBI motivational interviewing-based brief intervention; *ISF* implementation and sustainment facilitation; *UC* usual care; bolded arrows represent hypothesized relationships; dashed arrows represent interactions and cross-level interactions that will be examined

In 2013, the National Institute on Drug Abuse (NIDA) sought to fund research that would advance understanding of how best to improve the integration of SUD treatment services within HIV/AIDS service delivery settings [18]. In 2014, NIDA funded a type 2 effectiveness–implementation hybrid trial called the substance abuse treatment to HIV Care (SAT2HIV) Project [19]. As shown in Fig. 1, Aim 1 of the SAT2HIV Project tests the effectiveness of a motivational interviewing-based brief intervention (MIBI) for substance use as an adjunct to usual care within AIDS service organizations (ASOs) as part of its multisite MIBI Experiment [20]. Aim 2 of the SAT2HIV Project tests the effectiveness of implementation and sustainment facilitation (ISF) as an adjunct to the Addiction Technology Transfer Center’s (ATTC) model for training staff in motivational interviewing as part of its ISF Experiment. The current paper describes the study protocol for the ISF Experiment and has been written in accordance with the SPIRIT guidelines [21, 22] (see Additional file 1). A cluster randomized design with staff randomized within clusters of ASOs was used to minimize the likelihood of contamination across study conditions. Importantly, although randomization was at the cluster level (i.e., organization level), our objective and hypotheses pertain to staff-level outcomes. The study protocol for the MIBI Experiment, also written in accordance with the SPIRIT guidelines, has been published separately [20]. With this background, we describe below the objective, design, and methods for the SAT2HIV Project’s ISF Experiment.

### Rationale for the ISF Experiment’s EBP, outcomes, and strategies

#### Rationale for the targeted EBP

The selection of motivational interviewing as the to-be-implemented EBP was based on several factors, including (a) research reviews supporting the effectiveness of motivational interviewing in reducing substance use [23–25], (b) the availability of psychometrically sound measures for assessing the extent to which motivational interviewing was implemented with adherence and competence [26], and (c) a research review suggesting that HIV care settings have been receptive to implementing motivational interviewing for HIV medication adherence [27].

#### Rationale for the primary outcomes

Proctor et al. [28] defined “implementation outcomes” as the effects of deliberate and purposeful actions to implement new treatments, practices, and services. However, our interest in comparing the effectiveness of the two strategies during the preparation phase, implementation phase, and sustainment phase of the exploration–preparation–implementation–sustainment (EPIS) continuum [29] required selection of unique preparation, implementation, and sustainment outcomes. Building on prior preparation research [30], days-to-proficiency was selected as the ISF experiment’s primary preparation outcome. Klein and Sorra’s implementation effectiveness construct (i.e., the consistency and quality of targeted organizational members’ use of an innovation) [31] was selected as the ISF experiment’s primary implementation outcome. Implementation effectiveness is important, given it has been hypothesized to be a function of implementation strategies and implementation climate [32–34]. Finally, building on sustainment research that has used raw units (e.g., number of staff trained, number of clients served) to operationalize sustainment outcomes [35], the raw unit of MIBIs delivered during the project’s sustainment phase was selected as the ISF experiment’s primary sustainment outcome.

#### Rationale for the strategies tested

Guidance for strategy selection was drawn from the research of Miller et al. [30], which experimentally compared strategies for training individuals in motivational interviewing. Relative to the other conditions examined (e.g., workshop training, workshop plus feedback, workshop plus coaching), the most effective condition for helping individuals demonstrate proficiency in motivational interviewing was the workshop training plus feedback plus coaching condition. Given its empirical support, each of these discrete strategies is encompassed within the overarching strategy of centralized technical assistance that ATTCs across the United States use in training individuals in motivational interviewing [36] (hereafter referred to as the ATTC strategy).

Although the staff-focused ATTC strategy is viewed as necessary for helping staff learn motivational interviewing, we argue that it may be insufficient on its own for optimizing the preparation, implementation, and sustainment processes. As such, we sought to identify an effective adjunct to the ATTC strategy. Each of the discrete strategies identified by Powell et al. [37] were considered as potential adjuncts to the ATTC strategy. Use of an improvement or implementation advisor was selected as the overarching strategy-to-be-tested, as Gustafson et al. [38] found that of the strategies compared, clinic-level coaching (i.e., use of an improvement advisor) was the best strategy for decreasing patient wait-time and increasing the number of new patients. In addition, six other discrete strategies (develop tools for quality improvement, organize implementation team meetings, identify and prepare champions, assess for readiness and identify barriers, conduct local consensus discussions, and conduct cyclical small tests of change) were packaged with the implementation advisor and branded together as the ISF strategy.

### The ISF Experiment’s Objective and Scientific Hypotheses

Testing the effectiveness of the ISF strategy as an adjunct to the ATTC strategy is the ISF Experiment’s key objective. Table 1 lists the planned scientific hypotheses for the ISF Experiment, which were guided by use of a decomposed-first strategy [39] that advocates for starting with moderation-focused hypotheses to avoid biases associated with conflated effects.

**Table 1:** Planned scientific hypotheses

Hypotheses	
H1	The positive relationship between the implementation and sustainment facilitation and staff time-to-proficiency will be moderated by
	H1a Staff’s motivational interviewing experience
	H1b Staff’s personal recovery status
	H1c Organization’s readiness for implementing change
	H1d Organization’s implementation climate
	H1e Organization’s leadership engagement
	H1f Organization’s tension for change
H2	The positive relationship between the implementation and sustainment facilitation and staff implementation effectiveness will be moderated by
	H2a Staff’s motivational interviewing experience
	H2b Staff’s personal recovery status
	H2c Organization’s readiness for implementing change
	H2d Organization’s implementation climate
	H2e Organization’s leadership engagement
	H2f Organization’s tension for change
H3	The positive relationship between the implementation and sustainment facilitation and staff level of sustainment will be moderated by
	H3a Staff’s motivational interviewing experience
	H3b Staff’s personal recovery status
	H3c Organization’s readiness for implementing change
	H3d Organization’s implementation climate
	H3e Organization’s leadership engagement
	H3f Organization’s tension for change
H4	Staff implementation effectiveness will mediate the relationship between staff time-to-proficiency and staff level of sustainment

## Methods

### Participants, interventions, and outcomes

#### Study setting

The ISF experiment was conducted in community-based ASOs (N = 39; i.e., clusters) located across the United States in 23 states and the District of Columbia. ASOs conduct HIV prevention efforts and provide medical and nonmedical case management services (e.g., retention in care, medication adherence, referral to social services and specialty treatment) to individuals living with HIV/AIDS. ASOs are distinct from HIV primary care organizations, which provide medical services including prescriptions for antiretroviral therapy (ART), CD4 T-lymphocyte testing, and HIV viral load testing [40].

#### Eligibility criteria

To be eligible to participate, an ASO (i.e., the cluster) had to (1) serve a minimum of 100 individuals living with HIV/AIDS per year, (2) have at least two case management staff who were willing to be trained in the MIBI for substance use (hereafter referred to as BI staff) [20], and (3) have at least one leadership staff (e.g., supervisor, manager, director) willing to help ensure BI staff were given sufficient time for project participation. There were no exclusion criteria.

#### Intervention: preparation, implementation, and sustainment strategies

As highlighted by Proctor et al. [41], despite the importance of providing full and precise descriptions of implementation strategies (i.e., the methods or techniques used to enhance the adoption, implementation, and sustainment of a clinical program or practice) used or tested, few studies provide adequate detail in their publications. Thus, Proctor et al.’ recommended guidelines were used to identify, define, and operationalize the ATTC strategy (see Table 2) and the ISF strategy (see Table 3) along six key dimensions: actor, actions, targets of the actions, temporality, implementation outcomes affected, and justification. Complementing Tables 2 and 3, dose (i.e., frequency and intensity) of the ATTC strategy and ISF strategy is detailed for each of the three project phases: preparation phase (see Table 4; see Additional File 2 for single page version), implementation phase (see Table 5; see Additional File 3 for single page version), and sustainment phase (see Table 6; see Additional File 4 for single page version).

**Table 2:** Specification overview of the multifaceted Addiction Technology Transfer Center (ATTC) strategy

Discrete implementation strategies Defining characteristic according to Proctor et al. [41]	Operational definition of key dimensions for each discrete implementation strategy						
	Actor(s)	Actions(s)	Target(s) of the action	Temporality	Dose	Targeted implementation outcome(s)	Justification
*A. Centralized technical assistance*: Develop and use a system to deliver technical assistance focused on implementation issues	Regional ATTC (e.g., Mid-America, Northwest, Northeast)	The overarching discrete implementation strategy that encompasses the other discrete implementation strategies listed below	2 BI staff per ASO	The initial kickoff meeting should be within 1 month of completing the exploration phase	See Tables 4, 5 and 6	Fidelity (i.e., proficiency and implementation effectiveness	[36, 42–44]
*B. Develop educational materials*: Develop and format guidelines, manuals, toolkits, and other supporting materials in ways that make it easier for stakeholders to learn about the innovation and for clinicians to learn how to deliver the clinical innovation	Regional ATTC	The Motivational Interviewing-Based Brief Intervention (MIBI) protocol manual, which provides information and knowledge about how the MIBI is intended to be implemented	2 BI staff per ASO	Finalization of educational materials (e.g., MIBI protocol manual) should be prior to the initial kickoff meeting	See Tables 4, 5 and 6	Fidelity (i.e., proficiency and implementation effectiveness	[45, 46]
*C. Develop and organize quality monitoring system:* Develop and organize systems and procedures that monitor clinical processes and/or outcomes for quality assurance and improvement	Regional ATTC	A Web-based system (sat2hivproject.org), that enables secure and efficient sharing of data relevant to the evidence-based practice (EBP) preparation and implementation process	2 BI staff per ASO	Finalization of quality monitoring systems (i.e., sat2hivproject.org) should be prior to the initial kickoff meeting	See Tables 4, 5 and 6	Fidelity (i.e., proficiency and implementation effectiveness)	[49–51]
*D. Develop tools for quality monitoring*: Develop, test, and introduce quality-monitoring tools with inputs (e.g., measures) specific to the innovation being implemented	Regional ATTC	The Independent Tape Rater Scale (ITRS), which enables reliable and valid rating of the extent to which BI staff deliver the EBP with fidelity	2 BI staff per ASO	Finalization of tools for quality monitoring (i.e., ITRS) should be prior to the initial kickoff meeting	See Tables 4, 5 and 6	Fidelity (i.e., proficiency and implementation effectiveness)	[26, 52, 53]
*E. Distribute educational materials*: Distribute educational materials (e.g., manuals) in person, by mail, and/or electronically.	Regional ATTC	Distribute professionally printed copies of the MIBI protocol manual to each BI staff	2 BI staff per ASO	Distribute at the workshop training	See Tables 4, 5 and 6	Fidelity (i.e., proficiency and implementation effectiveness)	[45, 46, 54]
*F. Conduct educational meetings*: Hold meetings targeted toward providers, administrators, other organizational stakeholders, and community, patient or consumer, and family stakeholders to teach them about the clinical innovation	Regional ATTC	In-person and Web-based meetings that enable direct interaction between the actors (ATTC) and targeted users of the EBP (BI staff)	2 BI staff per ASO	Educational meetings should begin at least 3 months before the implementation phase begins	See Tables 4, 5 and 6	Fidelity (i.e., proficiency and implementation effectiveness)	[30, 36, 55, 61]
*G. Make training dynamic*: Vary the information delivery methods to cater to different learning styles and work contexts and shape the training in the innovation to be interactive	Regional ATTC	Incorporate standardized role plays that enable EBP trainees (BI staff) to practice with each other and that facilitate understanding of the EBP from both staff and client perspectives	2 BI staff per ASO	Should begin during the first contact	See Tables 4, 5 and 6	Fidelity (i.e., proficiency and implementation effectiveness)	[55, 56, 101, 102]
*H. Audit and provide feedback*: Collect and summarize clinical performance data over a specified period, and give data to clinicians and administrators in the hopes of changing provider behavior.	Regional ATTC	Generate and email standardized feedback reports to EBP trainees (BI staff) using the standardized quality monitoring tool (ITRS)	2 BI staff per ASO	Should begin approximately 1–2 weeks following the end of the in-person educational training workshop	See Tables 4, 5 and 6	Fidelity (i.e., proficiency and implementation effectiveness)	[30, 57–60]
*I. Provide ongoing consultation*: Provide clinicians with continued consultation with an expert in the clinical innovation	Regional ATTC	Phone-based individualized meetings that enable direct contact between the actor (ATTC trainer) and one EBP trainee (BI staff)	2 BI staff per ASO	Should begin approximately 1–2 weeks following the end of the in-person educational training workshop	See Tables 4, 5 and 6	Fidelity (i.e., proficiency and implementation effectiveness)	[30, 36, 61]
*J. Create a learning collaborative*: Develop and use groups of providers or provider organizations that will implement the clinical innovation and develop ways to learn from one another to foster better implementation	Regional ATTC	Web-based group meetings that enable direct contact between the actor (ATTC trainer) and a group (10–14 targeted users of the EBP, BI staff), who can share lessons learned	2 BI staff per ASO	Should begin approximately 3–4 weeks after the implementation phase begins	See Tables 4, 5 and 6	Fidelity (i.e., proficiency and implementation effectiveness)	[63–65]

**Table 3:** Specification overview of the multifaceted implementation and sustainment facilitation (ISF) Strategy

Discrete implementation strategies: Defining characteristic according to Proctor et al. [41]	Operational definition of key dimensions for each discrete implementation strategy						
	Actor(s)	Actions(s)	Target(s) of the action	Temporality	Dose	Targeted implementation outcome(s)	Justification
*K. Use an improvement and implementation advisor*: Seek guidance from experts in implementation, including consultation with outside experts (e.g., university-affiliated faculty members, quality improvement experts, implementation professionals)	An individual with training and experience in assisting organizations with practice improvement and implementation efforts	The overarching implementation strategy that encompasses the other discrete implementation strategies listed below	An ASO’s designated staff working on the project (SWOP) team (2 BI staff and 2–4 leadership staff) Implementation readiness, implementation climate, leadership engagement	The initial kickoff meeting should be held within 1 month of completing the exploration phase	See Tables 4, 5 and 6	Fidelity (i.e., proficiency and implementation effectiveness) and sustainment	[38, 66–69]
*L. Develop tools for quality improvement*: Develop, test, and introduce quality-improvement tools with inputs (e.g., measures) specific to the innovation being implemented	An individual with training and experience in assisting organizations with practice improvement and implementation efforts	Decisional Balance Exercise; Performance Review, Evaluation, and Planning Exercise; Climate Evaluation and Optimization Exercise	SWOP team. Implementation readiness, implementation climate, leadership engagement	Finalization of tools for quality improvement (e.g., decisional balance worksheet) should be prior to the initial kickoff meeting	See Tables 4, 5 and 6	Fidelity (i.e., proficiency and implementation effectiveness) and sustainment	[31, 38, 72, 103, 104]
*M. Organize implementation team meetings*: Develop and support teams of clinicians who are implementing the innovation and give them protected time to reflect on the implementation effort, share lessons learned, and support one another’s learning	An individual with training and experience in assisting organizations with practice improvement and implementation efforts	Meetings that enable direct interaction between the actors—implementation and sustainment facilitation (ISF) staff— and SWOP team	SWOP team. Implementation readiness, implementation climate, leadership engagement	First implementation team meeting should be held within 1 month of completing the exploration phase	See Tables 4, 5 and 6	Fidelity (i.e., proficiency and implementation effectiveness) and sustainment	[74, 75]
*N. Identify and prepare champions*: Cultivate relationships with people who will champion the clinical innovation and spread the word of the need for it	An individual with training and experience in assisting organizations with practice improvement and implementation efforts	Learning about and engaging with the SWOP team	SWOP team. Implementation readiness, implementation climate, leadership engagement	Identification and preparation of champions should begin during the process of organizing the initial implementation team meeting	See Tables 4, 5 and 6	fidelity (i.e., proficiency and implementation effectiveness) and sustainment	[31, 32, 76, 77]
*O. Assess for readiness and identify barriers*: Assess various aspects of an organization to determine its degree of readiness to implement, barriers that may impede implementation, and strengths that can be used in the implementation effort	An individual with training and experience in assisting organizations with practice improvement and implementation efforts	Utilization of the ISF exercises described above (L. Develop tools for quality improvement)	SWOP team. Implementation readiness, implementation climate, leadership engagement	Assessments of readiness and identification of barriers should begin during the process of organizing the initial implementation team meeting	See Tables 4, 5 and 6	Fidelity (i.e., proficiency and implementation effectiveness) and sustainment	[78–82]
*P. Conduct local consensus discussions*: Include providers and other stakeholders in discussions that address whether the chosen problem is important and whether the clinical innovation to address it is appropriate	An individual with training and experience in assisting organizations with practice improvement and implementation efforts	Completion of an in-person, stakeholder-engagement and sustainment-planning meeting	SWOP team. Implementation readiness, implementation climate, leadership engagement	Should be held as soon as possible after the first implementation month has been completed	See Tables 4, 5 and 6	Fidelity (i.e., proficiency and implementation effectiveness) and Sustainment	[83, 84, 105]
*Q. Conduct cyclical small tests of change*: Implement changes in a cyclical fashion using small tests of change	An individual with training and experience in assisting organizations with practice improvement and implementation efforts	Completion of study-act-plan-do cycles	SWOP team. Implementation readiness, implementation climate, leadership engagement	Should begin as soon as necessary	See Tables 4, 5 and 6	Fidelity (i.e., proficiency and implementation effectiveness) and sustainment	[85–87]

**Table 4:** Dose for each overarching strategy during the preparation phase (months 1–6)

Blended Strategy and the discrete strategies that it encompasses	Month 1			Month 2			Month 3		
	Training, coaching, or facilitation staff	ASO’s leadership staff	ASO’s BI staff	Training, coaching, or facilitation staff	ASO’s leadership staff	ASO’s BI staff	Training, coaching, or facilitation staff	ASO’s leadership staff	ASO’s BI staff
*Addiction Technology Transfer Center (ATTC)*									
A. Centralized technical assistance	As needed	NA	NA	As needed	NA	NA	As needed	NA	5 h
B. Develop educational materials	+			+					
C. Develop and organize quality monitoring systems	+			+					
D. Develop tools for quality monitoring	+			+					
E. Distribute educational materials							+		+
F. Conduct educational meetings							+		+
G. Make training dynamic							+		+
H. Audit and Provide feedback									
I. Provide ongoing consultation									
J. Create a learning collaborative									
*Implementation and sustainment facilitation (ISF)*									
K. Use an improvement and implementation advisor	As needed	NA	NA	As needed	As needed	As needed	1 h	1 h	1 h
L. Develop tools for quality improvement	+								
M. Organize implementation team meetings				+	+	+	+	+	+
N. Identify and prepare champions				+	+	+	+	+	+
O. Assess for readiness and identify barriers							+	+	+
P. Conduct local consensus discussions									
Q. Conduct cyclical small tests of change									

Blended Strategy and the discrete strategies that it encompasses	Month 4			Month 5			Month 6		
	Training, coaching, or facilitation staff	ASO’s leadership staff	ASO’s BI staff	Training, coaching, or facilitation staff	ASO’s leadership staff	ASO’s BI staff	Training, coaching, or facilitation staff	ASO’s leadership staff	ASO’s BI staff
*Addiction Technology Transfer Center (ATTC)*									
A. Centralized technical assistance	16 h	NA	16 h	As needed	NA	2–4 h	As needed	NA	2–4 h
B. Develop educational materials									
C. Develop and organize quality monitoring systems									
D. Develop tools for quality monitoring									
E. Distribute educational materials	+		+						
F. Conduct educational meetings	+		+						
G. Make training dynamic	+		+						
H. Audit and Provide feedback				+		+	+		+
I. Provide ongoing consultation				+		+	+		+
J. Create a learning collaborative									
*Implementation and sustainment facilitation (ISF)*									
K. Use an improvement and implementation advisor	1 h	1 h	1 h	1 h	1 h	1 h	1 h	1 h	1 h
L. Develop tools for quality improvement									
M. Organize implementation team meetings	+	+	+	+	+	+	+	+	+
N. Identify and prepare champions	+	+	+	+	+	+	+	+	+
O. Assess for readiness and identify barriers	+	+	+	+	+	+	+	+	+
P. Conduct local consensus discussions									
Q. Conduct cyclical small tests of change									

During the 6-month preparation phase, the ATTC strategy’s overarching discrete strategy (centralized technical assistance) encompasses 8 discrete strategies. During the 6-month preparation phase, the ISF strategy’s overarching discrete strategy (use an improvement and implementation advisor) encompasses 4 discrete strategies. For each month, intensity (i.e., time) is reported for the overarching strategy, with “+” being used to indicate the discrete strategies encompassed for that month

*NA* not applicable, *ASO* AIDS service organization, *BI* brief intervention

**Table 5:** Dose for each overarching strategy during the implementation phase (months 7–12)

Blended Strategy and the discrete strategies that it encompasses	Month 1			Month 2			Month 3		
	Training, coaching, or facilitation staff	ASO’s leadership staff	ASO’s BI staff	Training, coaching, or facilitation staff	ASO’s leadership staff	ASO’s BI staff	Training, coaching, or facilitation staff	ASO’s leadership staff	ASO’s BI staff
*Addiction Technology Transfer Center (ATTC)*									
A. Centralized technical assistance	As needed	NA	NA	As needed	NA	NA	As needed	NA	5 h
B. Develop educational materials									
C. Develop and organize quality monitoring systems									
D. Develop tools for quality monitoring									
E. Distribute educational materials									
F. Conduct educational meetings									
G. Make training dynamic									
H. Audit and Provide feedback	+		+	+		+	+		+
I. Provide ongoing consultation									
J. Create a learning collaborative	+		+	+		+	+		+
*Implementation and sustainment facilitation (ISF)*									
K. Use an improvement and implementation advisor	1 h	1 h	1 h	1 h	1 h	1 h	1 h	1 h	1 h
L. Develop tools for quality improvement									
M. Organize implementation team meetings	+	+	+	+	+	+	+	+	+
N. Identify and prepare champions	+	+	+	+	+	+	+	+	+
O. Assess for readiness and identify barriers	+	+	+	+	+	+	+	+	+
P. Conduct local consensus discussions				+	+	+			
Q. Conduct cyclical small tests of change				+	+	+	+	+	+

Blended Strategy and the discrete strategies that it encompasses	Month 4			Month 5			Month 6		
	Training, coaching, or facilitation staff	ASO’s leadership staff	ASO’s BI Staff	Training, coaching, or facilitation staff	ASO’s leadership staff	ASO’s BI staff	Training, coaching, or Facilitation Staff	ASO’s leadership Staff	ASO’s BI staff
*Addiction Technology Transfer Center (ATTC)*									
A. Centralized technical assistance	16 h	NA	16 h	As needed	NA	2-4 h	As needed	NA	2-4 h
B. Develop educational materials									
C. Develop and organize quality monitoring systems									
D. Develop tools for quality monitoring									
E. Distribute educational materials									
F. Conduct educational meetings									
G. Make training dynamic									
H. Audit and Provide feedback	+		+	+		+	+		+
I. Provide ongoing consultation									
J. Create a learning collaborative	+		+	+		+	+		+
*Implementation and sustainment facilitation (ISF)*									
K. Use an improvement and implementation advisor	1 h	1 h	1 h	1 h	1 h	1 h	1 h	1 h`	1 h
L. Develop tools for quality improvement									
M. Organize implementation team meetings	+	+	+	+	+	+	+	+	+
N. Identify and prepare champions	+	+	+	+	+	+	+	+	+
O. Assess for readiness and identify barriers	+	+	+	+	+	+	+	+	+
P. Conduct local consensus discussions									
Q. Conduct cyclical small tests of change	+	+	+	+	+	+	+	+	+

During the 6-month implementation phase, the ATTC strategy’s overarching discrete strategy (centralized technical assistance) encompasses 2 discrete strategies. During the 6-month implementation phase, the ISF strategy’s overarching discrete strategy (use an improvement and implementation advisor) encompasses 5 discrete strategies. For each month, intensity (i.e., time) is reported for the overarching strategy, with “+” being used to indicate the discrete strategies encompassed for that month

*NA* not applicable, *ASO* AIDS service organization, *BI* brief intervention

**Table 6:** Dose for each overarching strategy during the sustainment phase (months 13–18)

Blended Strategy and the discrete strategies that it encompasses	Month 1			Month 2			Month 3		
	Training, coaching, or facilitation staff	ASO’s leadership staff	ASO’s BI staff	Training, coaching, or facilitation staff	ASO’s leadership staff	ASO’s BI staff	Training, coaching, or facilitation staff	ASO’s leadership staff	ASO’s BI staff
*Addiction Technology Transfer Center (ATTC)*									
A. Centralized technical assistance	NA	NA	NA	NA	NA	NA	NA	NA	NA
B. Develop educational materials									
C. Develop and organize quality monitoring systems									
D. Develop tools for quality monitoring									
E. Distribute educational materials									
F. Conduct educational meetings									
G. Make training dynamic									
H. Audit and Provide feedback									
I. Provide ongoing consultation									
J. Create a learning collaborative									
*Implementation and sustainment facilitation (ISF)*									
K. Use an improvement and implementation advisor	1 h	1 h	1 h	1 h	1 h	1 h	1 h	1 h	1 h
L. Develop tools for quality improvement	+	+	+	+	+	+	+	+	+
M. Organize implementation team meetings	+	+	+	+	+	+	+	+	+
N. Identify and prepare champions	+	+	+	+	+	+	+	+	+
O. Assess for readiness and identify barriers	+	+	+	+	+	+	+	+	+
P. Conduct local consensus discussions	+	+	+	+	+	+	+	+	+
Q. Conduct cyclical small tests of change	+	+	+	+	+	+	+	+	+

Blended strategy and the discrete strategies that it encompasses	Month 4			Month 5			Month 6		
	Training, coaching, or facilitation staff	ASO’s leadership staff	ASO’s BI staff	Training, coaching, or facilitation staff	ASO’s leadership staff	ASO’s BI staff	Training, coaching, or facilitation staff	ASO’s leadership staff	ASO’S BI staff
*Addiction Technology Transfer Center (ATTC)*									
A. Centralized technical assistance	NA	NA	NA	NA	NA	NA	NA	NA	NA
B. Develop educational materials									
C. Develop and organize quality monitoring systems									
D. Develop tools for quality monitoring									
E. Distribute educational materials									
F. Conduct educational meetings									
G. Make training dynamic									
H. Audit and Provide feedback									
I. Provide ongoing consultation									
J. Create a learning collaborative									
*Implementation and sustainment facilitation (ISF)*									
K. Use an improvement and implementation advisor	1 h	1 h	1 h	1 h	1 h	1 h	1 h	1 h	1 h
L. Develop tools for quality improvement	+	+	+	+	+	+	+	+	+
M. Organize implementation team meetings	+	+	+	+	+	+	+	+	+
N. Identify and prepare champions	+	+	+	+	+	+	+	+	+
O. Assess for readiness and identify barriers	+	+	+	+	+	+	+	+	+
P. Conduct local consensus discussions	+	+	+	+	+	+	+	+	+
Q. Conduct cyclical small tests of change	+	+	+	+	+	+	+	+	+

The ATTC strategy’s is no longer provided during the 6-month sustainment phase. The ISF strategy is optional during the 6-month sustainment phase. Consistent with an organization-centered approach, any of the discrete strategies are options to use. However, if used the intensity (i.e., time) of the ISF strategy is limited to 1 h per month

*NA* not applicable, *ASO* AIDS service organization, *BI* brief intervention

### Addiction Technology Transfer Center strategy

Although the ATTC strategy has been used in addiction treatment settings, its use in HIV/AIDS service delivery settings is novel and thus one of the project’s innovations. The ATTC strategy represents a “blended strategy,” the term reserved for instances in which several discrete strategies are packaged together and protocolized or branded [37]. Centralized technical assistance is the overarching strategy of the ATTC strategy. Encompassed within the ATTC strategy are an additional nine discrete strategies. Descriptions of each, which supplement the specifications provided as part of Table 2, are provided here.

(A) Centralized technical assistance Consistent with prior research [36, 42–44], centralized technical assistance was operationalized as an individualized, hands-on approach to building an entity’s capacity for quality implementation of innovations. Squires et al. [36] successfully used this strategy to implement contingency management in substance use disorder treatment organizations.
(B) Develop educational materials Educational materials, such as intervention manuals, have been found to be useful for learning [45, 46]. Thus, we developed an online introduction to motivational interviewing course [47] and a training manual for the MIBI protocol [48].
(C) Develop and organize quality monitoring system Building on prior research [49–51], a Web-based quality monitoring system was developed. Key functions of this system were: (a) secure uploads of session recordings by BI staff, (b) efficient adherence and competence rating of session recordings by trained raters, (c) automated sending of session quality rating feedback to BI staff, and (d) generation of custom summary reports (e.g., by organization, by month) of session quality ratings.
(D) Develop tools for quality monitoring The Independent Tape Rater Scale (ITRS) [26, 52, 53] was developed and validated for monitoring the level of adherence and competence of 10 core motivational interviewing skills (e.g., open-ended questions, reflective statements, fostering collaboration).
(E) Distribute educational materials Consistent with research supporting the importance of using multiple dissemination strategies [45, 46, 54], the educational materials were distributed to BI staff. BI staff were emailed links to the online educational course [47] and printed copies of the MIBI protocol manual [48] were hand-delivered to staff at the in-person workshop training.
(F) Conduct educational meetings Research has not found educational materials by themselves to be sufficient for learning motivational interviewing [30, 55]. Thus, Web-based and in-person educational meetings were also provided, including a two-day in-person workshop training for BI staff on the MIBI protocol.
(G) Make training dynamic Role plays that enable trainees to practice with other trainees and facilitate understanding of the EBP from both the staff and client perspectives have been found to make motivational interviewing training more dynamic [30, 55, 56]. In addition to using role plays multiple times during the in-person workshop training, trainees were given role plays to complete during the week after the workshop training.
(H) Audit and provide feedback There is support for audit and feedback as an effective strategy, both in general [57–60] and specifically with learning motivational interviewing [30]. Thus, standardized feedback reports based on ratings using the validated Independent Tape Rater Scale [26] were provided to BI staff for all sessions completed and recorded.
(I) Provide ongoing consultation Providing ongoing consultation following workshop training has been supported as an important strategy to facilitate learning of psychosocial interventions [30, 36, 61]. During the 10-week post-workshop-training practice period, each trainee was allowed up to four individual consultation sessions with a member of the Motivational Interviewing Network of Trainers (MINT) [62].
(J) Create a learning collaborative The use of a learning collaborative has been identified as an important method of learning [63–65]. Thus, each month during the 6-month implementation phase, a motivational interviewing expert from MINT [62] organized and moderated two 1-h learning collaborative meetings, one for the ATTC only condition and one for the ATTC plus ISF condition.

### Implementation and sustainment facilitation strategy

Encompassed within the overarching strategy of using an implementation advisor are six additional discrete strategies. Supplementing the specifications in Table 3, descriptions of each of these strategies are provided here.

(K) Use an improvement/implementation advisor Consistent with prior research [38, 66–69], use of an implementation advisor was operationalized as an individual external to the organization who utilized interactive problem-solving and support to help the organization identify and achieve improvement and implementation goals.
(L) Develop tools for quality improvement Five quality improvement tools were developed and are described below.

First, the past implementation effort exercise, based on research emphasizing the importance of using past performance to improve future practice [70], was developed to facilitate organizations sharing with their advisor a past experience implementing an innovation. In addition to describing the past effort, organizations discussed the extent to which the effort was ultimately successful, unsuccessful, or had mixed results. Advisors used reflective listening skills to highlight the importance of the organization’s past implementation effort and how learning from the past may help them successfully achieve the goals of the current project’s preparation, implementation, and sustainment phases.

Next, the decisional-balance exercise was developed based on supporting research [71] and sought to evoke reasons behind the organization’s decision to implement the MIBI for substance use and to identify potential barriers.

Third, the ISF Workbook (a Microsoft Excel-based electronic workbook) was developed to standardize ISF strategy implementation, the lack of standardization having been a criticism of many implementation studies [41]. The ISF Workbook has five worksheets: (1) a project charter worksheet that lists the project’s goals, staff working on the project (SWOP) team members, and the implementation advisor’s name and contact info; (2) a meeting attendees and notes worksheet with a placeholder for documenting the date of all expected ISF meetings, the SWOP team members that attended each meeting, summary notes from the meeting, and a link to the meeting recording; (3) a preparation phase worksheet that includes the goals of the preparation phase and the ISF strategy’s performance review, evaluation, and planning exercise; (4) an implementation phase worksheet that includes the goals of the implementation phase and the ISF strategy’s performance review, evaluation, and planning exercise; and (5) a sustainment phase worksheet that includes a placeholder for entering what (if anything) the organization chooses as their goal(s) for the sustainment phase and the ISF strategy’s performance review, evaluation, and planning exercise.

Next, the process walk through exercise was developed based on prior research that has found walking through the steps of a process to be a helpful quality improvement tool [38, 72]. The process walk through exercise was conducted by having the SWOP team review a detailed process flow diagram with the following four key questions emphasized throughout the exercise: What is working well? What needs improvement? What is the plan for improving what needs improvement? What is the plan for maintaining what is working well? Although time was spent on what was working well and the plans for maintaining what was working well, explicit emphasis was on identifying what needs improvement and plans to enact improvements.

Last, the implementation climate evaluation exercise was developed to standardize an advisor’s process of evaluating the implementation climate for the MIBI (i.e., the extent to which it is expected and supported). Implementation climate has been hypothesized as a key mechanism of change for implementation strategies’ impact on implementation effectiveness [31–33, 73]. When there was no consensus on the implementation climate or when the implementation climate was poor, the ISF advisor sought to evoke reasons for the current beliefs, find ways to better align staff members’ beliefs, and develop plans to optimize the implementation climate. In contrast, when there was consensus on the implementation climate or when the implementation climate was strong, advisors facilitated discussion around maintaining or improving it.

(M) Organize implementation team meetings Organizing implementation team meetings that SWOP team members were willing and able to regularly attend was one of the most important strategies [74, 75]. ISF advisors sought to organize recurring implementation team meetings early in the process. Monthly team meetings were conducted via join.me, a collaboration tool with advanced phone conference and screening sharing capabilities. In addition, a limited number of in-person team meetings (typically just one) were organized for a day during the second month of the implementation phase.
(N) Identify and prepare champions Consistent with research highlighting the importance of having someone championing the organization’s implementation efforts [31, 32, 76, 77], an ISF advisor’s focus on champion identification began immediately upon formal introduction to the organization and its SWOP team. The ISF advisor paid attention to the extent SWOP team members responded to emails and meeting discussions as a way of identifying team members’ levels of engagement and team influence. Once an ISF advisor identified a potential champion, they sought to optimize the individual’s commitment to the project and its goals.
(O) Assess for readiness and identify barriers Building on extant research on readiness assessment and barrier identification [78–82], the ISF strategy included exercises developed to assist with assessing readiness and identifying barriers (e.g., past implementation effort exercise, decisional balance exercise, process walk through exercise), which were described earlier (see Develop tools for quality improvement).
(P) Conduct local consensus discussions Consensus-building is an important strategy [83, 84]. Thus, concerted efforts were directed towards conducting local consensus discussions with key stakeholders, who are internal or external individuals that the SWOP team considered key to directly and/or indirectly helping sustain the MIBI services over time. Key stakeholders were invited to attend the in-person ISF meeting to learn about the project and participate in a formal sustainment planning discussion.
(Q) Conduct cyclical small tests of change Cyclical small tests of change, such as plan-do-study-act cycles are a valuable quality improvement strategy [85–87]. Within the ISF strategy, however, this cycle was reframed into a study-act-plan-do cycle. This reframing was done to emphasize the importance of beginning with the study phase by assessing existing performance and then deciding about the need to act (or not act). When action or change was deemed necessary, a plan was developed and then implemented in the do phase.

### Outcomes

Table 7 describes the three staff-level outcome measures (i.e., time-to-proficiency, implementation effectiveness, and level of sustainment) used to examine the extent to which the ISF strategy serves as an effective adjunct to the ATTC strategy. Additionally, Table 7 describes the two staff-level measures (i.e., personal recovery status and motivational interviewing experience) and four organizational-level measures (i.e., readiness for implementing change, implementation climate, leadership engagement, and tension for change) that have been hypothesized as moderators of the relationship between organizational condition assignment (ATTC vs. ATTC + ISF) and each respective primary outcome measure.

**Table 7:** Instruments, instrument-related procedures, and measures

Measurement instrument	Measurement instrument time points and procedures					
	Staff survey #1 (t = month 0)	Preparation phase assessment (t = months 1–6)	Implementation phase assessment (t = months 7–12)	Staff survey #2 (t = month 13)	Staff survey #3 (t = month 19)	Measure name (purpose) Description
Independent Tape Rater Scale [26]		X^a^				Time to proficiency (preparation outcome) A continuous staff measure representing the number of days between completion of the workshop training by BI staff and their demonstration of proficiency [26, 30] in the motivational interviewing-based brief intervention (MIBI)
Independent Tape Rater Scale [26]			X^b^			Implementation effectiveness [31] (implementation outcome) A continuous staff measure representing the sum of the standardized cumulative number of MIBIs delivered by BI staff (i.e., consistency) and the cumulative integrity score of their delivery of the MIBI (i.e., quality)
Staff survey	X^c^			X^c^	X^c^	Level of sustainment (sustainment outcome) A continuous staff measure (no specified range) that represents the number of MIBIs a BI staff self-reports having delivered during the past 6 months
Staff survey	X^c^			X^c^	X^c^	Motivational Interviewing Experience (moderator variable) A staff measure indicating a BI staff’s perception of their motivational interviewing experience (0 = none, 1 = beginner, 2 = intermediate, 3 = advanced, 4 = expert)
Staff survey	X^c^			X^c^	X^c^	Readiness for implementing change (moderator variable) A continuous organization measure (ranges from 1 to 5) that represents the organizational average of 6 items, each rated on a 5-point scale (1 = disagree, 2 = somewhat disagree, 3 = neither agree nor disagree, 4 = somewhat agree, 5 = agree) and adapted from Shea et al.’ readiness measure [106]
Staff survey	X^c^			X^c^	X^c^	Implementation climate (moderator variable) A continuous organization measure (ranges from 1 to 5) that represents the organizational average of 6 items, each rated on a 5-point scale (1 = disagree, 2 = somewhat disagree, 3 = neither agree nor disagree, 4 = somewhat agree, 5 = agree) and adapted from Jacobs et al.’ implementation measure [107]
Staff survey	X^c^			X^c^	X^c^	Leadership engagement (moderator variable) A continuous organization measure (ranges from 0 to 6) that represents the organizational average of 4 items, each rated on a 7-point scale (0 = not at all to 6 = highest extent possible) and developed for this study based on the leadership engagement construct described by Damschroder et al.—commitment, involvement, and accountability of leaders with the implementation [108]
Staff Survey	X^c^			X^c^	X^c^	Tension for change (moderator variable) A continuous organization measure (ranges from 0 to 6) that represents the organizational average of 3 items, each rated on a 7-point scale (0 = not at all to 6 = highest extent possible) and developed for this study based on the tension for change construct described by Damscroder et al.—stakeholders’ shared perception of the extent to which a change is important, needed, and desired [108]

^a^Ratings during the preparation phase were completed by one of the co-authors (DV)

^b^Ratings during the implementation phase were rated by one of 15 experienced raters, each of which were trained, calibrated, and monitored by one of the co-authors (SM)

^c^Staff surveys (electronically administered) required approximately 30 min to complete, and staff received one $25 e-gift card per completed survey as compensation

### Participant timeline

**Fig. 2:**
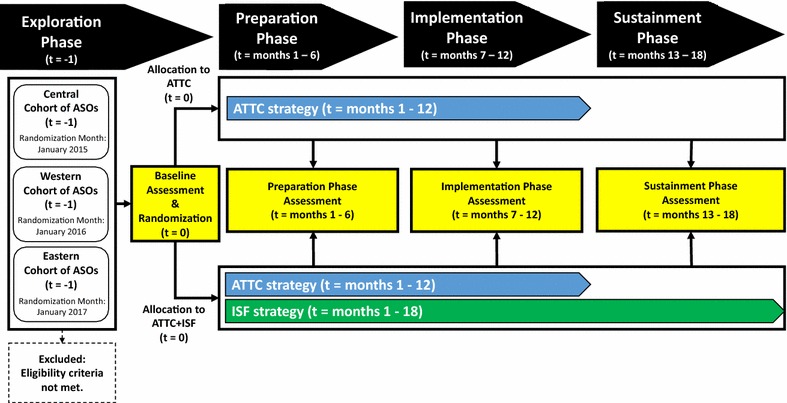
Flow of participating AIDS service organizations (ASOs) *Note*: *t* time; *ATTC* Addiction Technology Transfer Center; *ISF* implementation and sustainment facilitation

Figure 2 depicts the participant flow for the ISF Experiment, which was organized by the four-phased EPIS framework [29]. For each of the three ASO cohorts, which were spaced 1 year apart, the exploration phase was initiated via the dissemination of standardized project introductions via emails and phone calls to all ASOs within the cohort’s geographically based catchment area (i.e., Central, Western, and Eastern states). ASOs interested in learning more about the project were invited to participate in an introductory meeting (see Recruitment below). Following the meeting, ASOs that met project eligibility criteria were emailed a project participation agreement to be completed and returned to the project’s Principal Investigator for finalizing. Once a cohort’s target number of participation agreements was reached, the exploration phase concluded by having each ASO’s designated SWOP team members (2–4 leadership staff and 2 BI staff) complete a confidential baseline assessment survey. As described in in the allocation section, data from these surveys, conducted under the auspices of RTI’s Institutional Review Board (IRB) and requiring written consent, were used as part of the condition assignment process. Following the completion of the exploration phase, ASOs and their SWOP team completed the project’s three 6-month phases: preparation (months 1–6), implementation (months 7–12), and sustainment (months 13–18).

#### Sample size

Sample size for the ISF Experiment was determined via power analyses with Optimal Design Software [88]. We assumed an equal number of BI staff (2 per ASO) and an intraclass correlation coefficient of .05. With 78 BI staff nested within 39 ASOs, there is 80% power to detect statistically significant (p < .05) differences when the effect size is .67 or greater.

#### Recruitment

The identification and recruitment of ASOs was conducted by the Principal Investigator (BG) and project coordinators (DK, EB). Potential ASOs were identified via searches of organization directories [89, 90]. Identified ASOs were sent standardized introduction emails, with follow-up calls completed as necessary by project coordinators. ASOs interested in learning more about the project participated in a 45- to 60-min, organization-specific, Web-assisted, informational webinar, which was conducted by the Principal Investigator or one of the project coordinators.

In addition to providing information about the project, a key goal of the informational webinar was to gather information about the ASO, including (a) whether describing their organization as a community-based ASO was accurate, (b) the key services provided to individuals living with HIV/AIDS, (c) the number of individuals living with HIV/AIDS served annually, (d) the number of case-management staff, (e) their level of interest in participating in the project, and (f) their reasons for wanting to participate. Upon review of the collected information, the Principal Investigator and project coordinators identified ASOs that did not represent a good fit for the project. ASOs deemed to be a good fit were contacted via email and/or phone, and official participation was documented by having the ASO’s signing official sign and date a project participation agreement.

### Assignment of interventions

#### Allocation

Participating ASOs were assigned to one of two study conditions via urn randomization [91]. Using staff survey data collected during the exploration phase from the BI staff and leadership staff, seven organizational-level factors (importance of substance use screening, importance of brief intervention for substance use, innovation-value fit, implementation strategy-value fit, implementation climate for substance use brief intervention, implementation readiness for substance use brief intervention, and implementation effectiveness for substance use brief intervention) were entered into the urn randomization program gRAND [92], which optimized the balance of the two study conditions on these seven factors. Written consent was obtained from BI staff and leadership staff before survey completion.

#### Blinding (masking)

ASOs and their staff were not blinded to study condition. However, the ATTC training and rating staff were blinded to study condition.

### Data collection, management, and analysis

#### Data collection and management

The Independent Tape Rater Scale (ITRS) was used to assess proficiency in motivational interviewing and implementation effectiveness. The ITRS is a well-validated tool for assessing two key factors: adherence and competence [26]. Confirmatory factor analysis has supported the two-factor structure of the ITRS [26], and excellent levels of inter-rater reliability have been found for both motivational interviewing adherence (mean ICC .89; range .66–.99) and competence (mean ICC .85; range .69–.97) [26].

The lead developer of the MIBI protocol (co-author SM) oversaw the selection, training, calibration, and supervision of the project’s 15 MIBI raters, who were blinded to study condition. Booster trainings and recalibration of the raters were conducted in between cohorts. Consistent with the established guidelines promoted in the motivational interviewing assessment: Supervisory Tools for Enhancing Proficiency [93], BI staff were considered to have demonstrated proficiency when at least half of the 10 motivational interviewing consistent items were rated 4 or greater on a 7-point scale for both adherence and competence. Submissions of MIBI sessions from BI staff and ratings from MIBI raters were enabled via a secure, Web-based implementation tracking system adapted from one used in our prior implementation research [94]. In addition to enabling MIBI raters to stream the audio files rather than download, an important security feature, the Web-based system allowed MIBI raters to enter adherence and competence ratings directly into the secure and backed-up database located on RTI’s servers.

As shown in Table 7, ASO staff participating in the ISF Experiment were invited to complete staff surveys at three time points: the exploration phase assessment at month zero, the implementation phase assessment at month 13, and the sustainment phase assessment at month 19. In addition to collecting background information for each participant (e.g., age, race, ethnicity, gender, educational level, tenure in profession, tenure with organization, salary, substance use recovery status), staff surveys assessed several domains theorized to be of importance—namely innovation values-fit, tension for change, implementation climate, implementation readiness, and leadership engagement—and assessed both the number of clients screened for substance use and the number clients to whom a brief intervention for substance use was delivered.

Given the professional level of ASO staff, surveys were self-administered. However, as a means of ensuring the highest quality data possible, surveys were Excel-based to help prevent common data quality issues like out-of-range responses. In addition to these real-time quality assurance measures, all staff surveys received quality assurance reviews from a project coordinator. When issues were identified, the project coordinator contacted the participant via email and/or phone to resolve the issue. Once the staff survey was complete, it was exported into a master database on one of RTI’s secure access-controlled servers that are backed up nightly. Each survey required about 30–45 min to complete, and participants received a $25 e-gift card as compensation for their time.

### Statistical methods

Statistical analyses will be conducted using an intention-to-treat analysis approach, which will analyze all ASOs as randomized. Hot-deck imputation [95, 96] will be used to address missing data issues, which are anticipated to be minimal (i.e., less than 5%). Analyses will be conducted using HLM software [97], which is well-suited for handling clustered data (i.e., time nested within staff, nested within organization). Analyses will be conducted in the order outlined in Table 1. In addition to reporting the coefficient, standard error, 95% confidence interval, and *p* value, results will also include effect size indicators.

### Monitoring

#### Data monitoring

The ISF Experiment was conducted under the auspices of RTI International’s IRB. The Principal Investigator of the ISF Experiment, however, assumes ultimate responsibility for the project’s data and safety monitoring.

#### Harms

Minimal risks are associated with the study and are limited to the potential breach of confidentiality. All adverse events are reported to the Principal Investigator within 24 h. Adverse events are reported to the IRB within 2 weeks of the Principal Investigator’s awareness of the event, with serious adverse events reported within 1 week.

#### Auditing

RTI International’s IRB conducts annual and random audits to assess adherence to federal human subjects protection regulations and to ensure that the rights and welfare of human subjects are protected.

### Ethics and dissemination

#### Research ethics approval

The ISF Experiment was reviewed and approved by RTI International’s IRB, under Federalwide Assurance No. 3331 from the Department of Health and Human Services’ Office for Human Research Protections.

#### Protocol amendments

Any protocol modification that may affect the conduct of the study, potential benefit to the participants, or participant safety requires a protocol amendment. All amendments were submitted to RTI International’s IRB for approval, with no protocol modification implemented until after notification of IRB approval.

#### Consent

In addition to having ASOs complete a project participation agreement, written consent was obtained from both leadership staff and BI staff. The project’s IRB-approved informed consent form was emailed to potential participants along with a password-protected assurance of consent form, the password for which was sent in a separate email. Individuals could not participate in the project without first completing the form.

#### Confidentiality

Information provided as part of the study is confidential and not shared with anyone outside the study. The exception, however, is if the participant has a plan to harm himself or herself or another specific person. Efforts to protect participant confidentiality include the following: (1) use of a unique participant ID number only accessible to the ASO study staff and a limited number of RTI study staff, (2) any study document (paper or electronic) that contains both the participant name and ID number is securely stored (e.g., locked file cabinet in a secure building, folder located on a password-protected server in a secure building), and (3) when study results are presented at meetings or published in journals, no identifying participant information will be included. Except for the assurance of consent form, which is required to be stored for at least 3 years after study completion, documents with identifying information will be destroyed within 90 days of study completion.

#### Declaration of interests

There are no competing interests or conflicts of interest to be declared.

#### Access to data

Access to data is restricted during the active data collection period and is limited to the Principal Investigator, data coordinators, statistician, and statistical programmer. Following the completion of the study, a public access dataset will be created and made available upon request to the Principal Investigator.

#### Ancillary and post-trial care

No ancillary or post-trial care is planned.

#### Dissemination policy

Irrespective of the magnitude or direction of effect, study findings will be disseminated. Dissemination efforts will include presentations at professional scientific conferences and publication in peer-reviewed journals. To the extent possible, we will seek to ensure study publications are open access (i.e., available online to readers without financial, legal, or technical barriers beyond those inseparable from gaining access to the internet).

## Discussion

In this paper, the study protocol for the SAT2HIV Project’s ISF Experiment, a cluster-randomized trial on the effectiveness of the ISF strategy as an adjunct to the ATTC strategy (Aim 2 of the parent SAT2HIV Project), has been described in accordance with the SPIRIT guidelines [21, 22]. In the sections below, we highlight and discuss: (1) key trial-relevant events (anticipated and unanticipated) that have occurred to date, (2) key limitations and strengths of the ISF Experiment, and (3) key anticipated impacts of the ISF Experiment.

### Trial-relevant events that have occurred to date

Table 8 summarizes key anticipated and unanticipated trial-relevant events that have occurred and that help illustrate the ISF Experiment’s progression and changing outer context.

**Table 8:** Key trial-relevant events to date

Calendar year	Calendar month	Project year	Project month	Key project-relevant events
2014	July	Year 1	Month 1	The grant received a $565,695 (16%) reduction in its total budget, which resulted in reducing the targeted number of participating organizations and dropping a specific aim on the cost-effectiveness of the ISF intervention
	August		Month 2	
	September		Month 3	
	October		Month 4	
	November		Month 5	The Principal Investigator (Dr. Garner) moved from Chestnut Health Systems to RTI International The grant was relinquished to the National Institute on Drug Abuse (NIDA)
	December		Month 6	
2015	January		Month 7	Cohort 1: Preparation phase initiated
	February		Month 8	The grant, minus the costs incurred during the first 5 months of the grant, was awarded to RTI International with Dr. Garner as the Principal Investigator
	March		Month 9	
	April		Month 10	
	May		Month 11	
	June		Month 12	Cohort 1: Preparation phase completed
	July	Year 2	Month 13	Cohort 1: Implementation phase initiated The updated United States National HIV/AIDS Strategy was released
	August		Month 14	
	September		Month 15	
	October		Month 16	
	November		Month 17	
	December		Month 18	Cohort 1: Implementation phase completed
2016	January		Month 19	Cohort 1: Sustainment phase initiated Cohort 2: Preparation phase initiated
	February		Month 20	
	March		Month 21	
	April		Month 22	
	May		Month 23	
	June		Month 24	Cohort 1: Sustainment phase completed Cohort 2: Preparation phase completed
	July	Year 3	Month 25	Cohort 2: Implementation phase initiated
	August		Month 26	
	September		Month 27	
	October		Month 28	
	November		Month 29	
	December		Month 30	Cohort 2: Implementation phase completed

*ISF* implementation and sustainment facilitation

### Key Limitations and Strengths of the ISF Experiment

The SAT2HIV Project’s ISF Experiment has limitations and strengths that are important to acknowledge. Key limitations include (1) the sustainment phase observation period being limited to 6 months, (2) the level of sustainment being limited to self-reports, and (3) cost-effectiveness not being examined. These limitations, however, are outweighed by the project’s many strengths.

Key strengths include the ISF Experiment’s (1) highly rigorous design as a randomized, hypothesis-driven experiment using psychometrically sound measures, (2) focus on the high-need setting of ASOs, (3) large sample size of 39 ASOs with 4-6 staff per ASO, (4) large geographic representation (23 states and the District of Columbia), and (5) examination of multiple phases of the EPIS continuum (preparation phase, implementation phase, and sustainment phase).

### Potential impacts of the ISF experiment

**Fig. 3:**
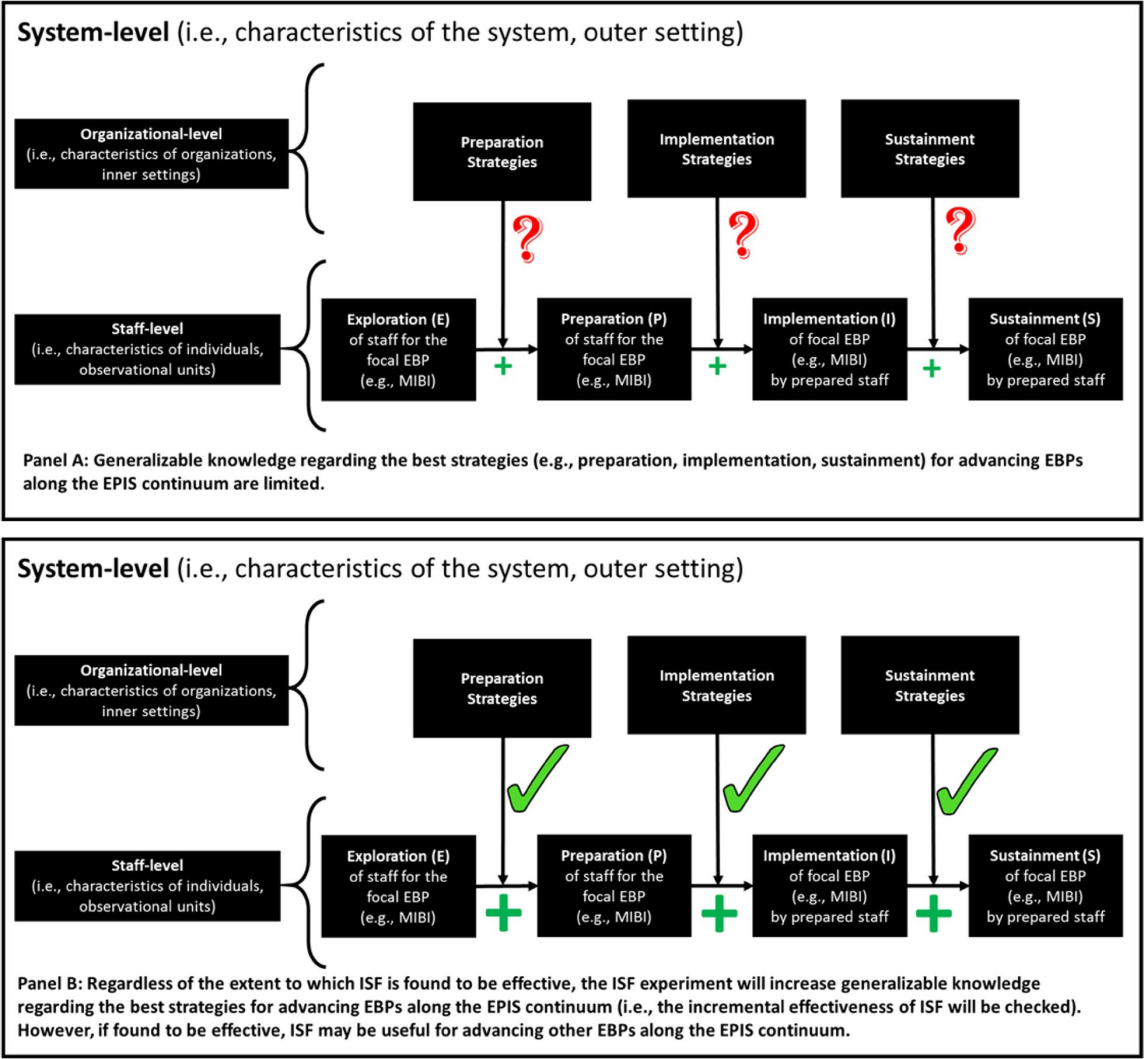
Potential impacts of the SAT2HIV Project’s ISF experiment

Panel A of Fig. 3 illustrates the current state of implementation research, where generalizable knowledge regarding the best approach for advancing EBPs along the EPIS continuum is limited, represented by question marks. Panel B of Fig. 3 illustrates that, regardless of the extent to which the ISF strategy is found to be an effective adjunct to the ATTC strategy, the ISF Experiment’s examination (represented by checkmarks) will increase generalizable knowledge regarding preparation, implementation, and sustainment strategies for advancing EBPs along the EPIS continuum. Beyond its impact on implementation research, the ISF Experiment may positively impact one or more key performance measures along the HIV Care Continuum (e.g., being linked to care, being engaged in care, being prescribed ART, achieving viral suppression). Indeed, the ISF Experiment may help advance ASO’s capacity to address substance use, which is important given that substance use has been shown to negatively impact being engaged in care, the most significant break point along the U.S. HIV Care Continuum [98–100].

## Conclusion

The SAT2HIV Project’s ISF Experiment represents one of the largest and most rigorous implementation research experiments to date. Nonetheless, should it support the ISF strategy as an effective adjunct to the ATTC strategy for implementing a motivational interviewing-based brief intervention for substance use within ASOs, future research must examine the extent to which study findings can be replicated, improved upon, and generalized to other contexts and EBPs. Our hope is that the ISF strategy is a replicable strategy that can be used to help improve public health advancing EBPs along the EPIS continuum.

## Additional files

**Additional file 1.** SPIRIT Checklist.

**Additional file 2.** Table 4.

**Additional file 3.** Table 5.

**Additional file 4.** Table 6.

## Abbreviations

AIDS: acquired immunodeficiency syndrome
ART: antiretroviral therapy
ASO: AIDS service organization
ATTC: Addiction Technology Transfer Center
BI: brief intervention
EBP: evidence-based practice
EPIS: exploration–preparation–implementation–sustainment
HIV: human immunodeficiency virus
IRB: Institutional Review Board
ISF: Implementation and sustainment facilitation
ITRS: Independent Tape Rater Scale
MIA: motivational interviewing assessment
MIBI: motivational interviewing-based brief intervention
NIDA: National Institute on Drug Abuse
SAT2HIV: substance abuse treatment to HIV Care
SPIRIT: Standard Protocol Items: Recommendations for Interventional Trials
SUD: substance use disorder
SWOP: staff working on the project
UC: usual care
UC + MIBI: usual care plus motivational interviewing-based brief intervention
