# Supplementary material for: Testing the implementation and sustainment facilitation (ISF) strategy as an effective adjunct to the Addiction Technology Transfer Center (ATTC) strategy: study protocol for a cluster randomized trial
Source: Addict Sci Clin Pract. 2017 Nov 17;12:32. doi: 10.1186/s13722-017-0096-7 (PMC5693537; doi:10.1186/s13722-017-0096-7)
Supplement: Supplementary file 2 — Additional file 2. Table 4. [file 13722_2017_96_MOESM2_ESM.pdf]

Table 4. Dose for Each Overarching Strategy During the Preparation Phase (Months 1 – 6)

		Month 1			Month 2			Month 3			Month 4			Month 5			Month 6		
	**Blended Strategy and the discrete strategies that it encompasses**	**Training, Coaching, or Facilitation Staff**	**ASO's Leadership Staff**	**ASO's BI Staff**	**Training, Coaching, or Facilitation Staff**	**ASO's Leadership Staff**	**ASO's BI Staff**	**Training, Coaching, or Facilitation Staff**	**ASO's Leadership Staff**	**ASO's BI Staff**	**Training, Coaching, or Facilitation Staff**	**ASO's Leadership Staff**	**ASO's BI Staff**	**Training, Coaching, or Facilitation Staff**	**ASO's Leadership Staff**	**ASO's BI Staff**	**Training, Coaching, or Facilitation Staff**	**ASO's Leadership Staff**	**ASO's BI Staff**
**ADDICTION TECHNOLOGY TRANSFER CENTER (ATTC)**	**A. Centralized technical assistance**	**As Needed**	**NA**	**NA**	**As Needed**	**NA**	**NA**	**As Needed**	**NA**	**5 Hours**	**16 Hours**	**NA**	**16 Hours**	**As Needed**	**NA**	**2-4 Hours**	**As Needed**	**NA**	**2-4 Hours**
	B. Develop educational materials	+			+														
	C. Develop and organize quality monitoring systems	+			+														
	D. Develop tools for quality monitoring	+			+														
	E. Distribute educational materials							+		+	+		+						
	F. Conduct educational meetings							+		+	+		+						
	G. Make training dynamic							+		+	+		+						
	H. Audit & Provide feedback													+		+	+		+
	I. Provide ongoing consultation													+		+	+		+
	J. Create a learning collaborative																		
**IMPLEMENTATION & SUSTAINMENT FACILITATION (ISF)**	**K. Use an improvement and implementation advisor**	**As Needed**	**NA**	**NA**	**As Needed**	**As Needed**	**As Needed**	**1 Hour**	**1 Hour**	**1 Hour**	**1 Hour**	**1 Hour**	**1 Hour**	**1 Hour**	**1 Hour**	**1 Hour**	**1 Hour**	**1 Hour**	**1 Hour**
	L. Develop tools for quality improvement	+																	
	M. Organize implementation team meetings				+	+	+	+	+	+	+	+	+	+	+	+	+	+	+
	N. Identify and prepare champions				+	+	+	+	+	+	+	+	+	+	+	+	+	+	+
	O. Assess for readiness and identify barriers							+	+	+	+	+	+	+	+	+	+	+	+
	P. Conduct local consensus discussions																		
	Q. Conduct cyclical small tests of change																		

Note: During the 6-month preparation phase, the ATTC strategy's overarching discrete strategy (centralized technical assistance) encompasses 8 discrete strategies. During the 6-month preparation phase, the ISF strategy's overarching discrete strategy (use an improvement and implementation advisor) encompasses 4 discrete strategies. For each month, intensity (i.e., time) is reported for the overarching strategy, with "+" being used to indicate the discrete strategies encompassed for that month. NA = not applicable; ASO = AIDS service organization; BI = brief intervention.
