# Supplementary material for: Testing the implementation and sustainment facilitation (ISF) strategy as an effective adjunct to the Addiction Technology Transfer Center (ATTC) strategy: study protocol for a cluster randomized trial
Source: Addict Sci Clin Pract. 2017 Nov 17;12:32. doi: 10.1186/s13722-017-0096-7 (PMC5693537; doi:10.1186/s13722-017-0096-7)
Supplement: Supplementary file 3 — Additional file 3. Table 5. [file 13722_2017_96_MOESM3_ESM.pdf]

Table 5. Dose for Each Overarching Strategy During the Implementation Phase (Months 7 – 12)

		Month 1			Month 2			Month 3			Month 4			Month 5			Month 6		
	**Blended Strategy and the discrete strategies that it encompasses**	**Training, Coaching, or Facilitation Staff**	**ASO's Leadership Staff**	**ASO's BI Staff**	**Training, Coaching, or Facilitation Staff**	**ASO's Leadership Staff**	**ASO's BI Staff**	**Training, Coaching, or Facilitation Staff**	**ASO's Leadership Staff**	**ASO's BI Staff**	**Training, Coaching, or Facilitation Staff**	**ASO's Leadership Staff**	**ASO's BI Staff**	**Training, Coaching, or Facilitation Staff**	**ASO's Leadership Staff**	**ASO's BI Staff**	**Training, Coaching, or Facilitation Staff**	**ASO's Leadership Staff**	**ASO's BI Staff**
**ADDICTION TECHNOLOGY TRANSFER CENTER (ATTC)**	**A. Centralized technical assistance**	**As Needed**	**NA**	**NA**	**As Needed**	**NA**	**NA**	**As Needed**	**NA**	**5 Hours**	**16 Hours**	**NA**	**16 Hours**	**As Needed**	**NA**	**2-4 Hours**	**As Needed**	**NA**	**2-4 Hours**
	B. Develop educational materials																		
	C. Develop and organize quality monitoring systems																		
	D. Develop tools for quality monitoring																		
	E. Distribute educational materials																		
	F. Conduct educational meetings																		
	G. Make training dynamic																		
	H. Audit & Provide feedback	+		+	+		+	+		+	+		+	+		+	+		+
	I. Provide ongoing consultation																		
	J. Create a learning collaborative	+		+	+		+	+		+	+		+	+		+	+		+
**IMPLEMENTATION & SUSTAINMENT FACILITATION (ISF)**	**K. Use an improvement and implementation advisor**	**1 Hour**	**1 Hour**	**1 Hour**	**1 Hour**	**1 Hour**	**1 Hour**	**1 Hour**	**1 Hour**	**1 Hour**	**1 Hour**	**1 Hour**	**1 Hour**	**1 Hour**	**1 Hour**	**1 Hour**	**1 Hour**	**1 Hour**	**1 Hour**
	L. Develop tools for quality improvement																		
	M. Organize implementation team meetings	+	+	+	+	+	+	+	+	+	+	+	+	+	+	+	+	+	+
	N. Identify and prepare champions	+	+	+	+	+	+	+	+	+	+	+	+	+	+	+	+	+	+
	O. Assess for readiness and identify barriers	+	+	+	+	+	+	+	+	+	+	+	+	+	+	+	+	+	+
	P. Conduct local consensus discussions				+	+	+												
	Q. Conduct cyclical small tests of change				+	+	+	+	+	+	+	+	+	+	+	+	+	+	+

Note: During the 6-month implementation phase, the ATTC strategy's overarching discrete strategy (centralized technical assistance) encompasses 2 discrete strategies. During the 6-month implementation phase, the ISF strategy's overarching discrete strategy (use an improvement and implementation advisor) encompasses 5 discrete strategies. For each month, intensity (i.e., time) is reported for the overarching strategy, with "+" being used to indicate the discrete strategies encompassed for that month. NA = not applicable; ASO = AIDS service organization; BI = brief intervention.
