# Supplementary material for: Testing the implementation and sustainment facilitation (ISF) strategy as an effective adjunct to the Addiction Technology Transfer Center (ATTC) strategy: study protocol for a cluster randomized trial
Source: Addict Sci Clin Pract. 2017 Nov 17;12:32. doi: 10.1186/s13722-017-0096-7 (PMC5693537; doi:10.1186/s13722-017-0096-7)
Supplement: Supplementary file 4 — Additional file 4. Table 6. [file 13722_2017_96_MOESM4_ESM.pdf]

Table 6. Dose for Each Overarching Strategy During the Sustainment Phase (Months 13 – 18)

		Month 1			Month 2			Month 3			Month 4			Month 5			Month 6		
	**Blended Strategy and the discrete strategies that it encompasses**	**Training, Coaching, or Facilitation Staff**	**ASO's Leadership Staff**	**ASO's BI Staff**	**Training, Coaching, or Facilitation Staff**	**ASO's Leadership Staff**	**ASO's BI Staff**	**Training, Coaching, or Facilitation Staff**	**ASO's Leadership Staff**	**ASO's BI Staff**	**Training, Coaching, or Facilitation Staff**	**ASO's Leadership Staff**	**ASO's BI Staff**	**Training, Coaching, or Facilitation Staff**	**ASO's Leadership Staff**	**ASO's BI Staff**	**Training, Coaching, or Facilitation Staff**	**ASO's Leadership Staff**	**ASO's BI Staff**
ADDICTION TECHNOLOGY TRANSFER CENTER (ATTC)	**A. Centralized technical assistance**	NA	NA	NA	NA	NA	NA	NA	NA	NA	NA	NA	NA	NA	NA	NA	NA	NA	NA
	B. Develop educational materials																		
	C. Develop and organize quality monitoring systems																		
	D. Develop tools for quality monitoring																		
	E. Distribute educational materials																		
	F. Conduct educational meetings																		
	G. Make training dynamic																		
	H. Audit & Provide feedback																		
	I. Provide ongoing consultation																		
	J. Create a learning collaborative																		
IMPLEMENTATION & SUSTAINMENT FACILITATION (ISF)	**K. Use an improvement and implementation advisor**	1 Hour	1 Hour	1 Hour	1 Hour	1 Hour	1 Hour	1 Hour	1 Hour	1 Hour	1 Hour	1 Hour	1 Hour	1 Hour	1 Hour	1 Hour	1 Hour	1 Hour	1 Hour
	L. Develop tools for quality improvement	+	+	+	+	+	+	+	+	+	+	+	+	+	+	+	+	+	+
	M. Organize implementation team meetings	+	+	+	+	+	+	+	+	+	+	+	+	+	+	+	+	+	+
	N. Identify and prepare champions	+	+	+	+	+	+	+	+	+	+	+	+	+	+	+	+	+	+
	O. Assess for readiness and identify barriers	+	+	+	+	+	+	+	+	+	+	+	+	+	+	+	+	+	+
	P. Conduct local consensus discussions	+	+	+	+	+	+	+	+	+	+	+	+	+	+	+	+	+	+
	Q. Conduct cyclical small tests of change	+	+	+	+	+	+	+	+	+	+	+	+	+	+	+	+	+	+

Note: The ATTC strategy's is no longer provided during the 6-month sustainment phase. The ISF strategy is optional during the 6-month sustainment phase. Consistent with an organization-centered approach, any of the discrete strategies are options to use. However, if used the intensity (i.e., time) of the ISF strategy is limited to 1 hour per month. NA = not applicable; ASO = AIDS service organization; BI = brief intervention.
